# A genome-wide association study for loin depth and muscle pH in pigs from intensely selected purebred lines

**DOI:** 10.1186/s12711-023-00815-0

**Published:** 2023-06-15

**Authors:** Suzanne Desire, Martin Johnsson, Roger Ros-Freixedes, Ching-Yi Chen, Justin W. Holl, William O. Herring, Gregor Gorjanc, Richard J. Mellanby, John M. Hickey, Melissa K. Jungnickel

**Affiliations:** 1 The Roslin Institute, The University of Edinburgh, Midlothian, UK; 2 Swedish University of Agricultural Sciences, Uppsala, Sweden; 3 Departament de Ciència Animal, Universitat de Lleida-Agrotecnio-CERCA Center, Lleida, Spain; 4 The Pig Improvement Company, Genus Plc, Hendersonville, TN USA; 5 The Royal (Dick) School of Veterinary Studies, The University of Edinburgh, Midlothian, UK

## Abstract

**Background:**

Genome-wide association studies (GWAS) aim at identifying genomic regions involved in phenotype expression, but identifying causative variants is difficult. Pig Combined Annotation Dependent Depletion (pCADD) scores provide a measure of the predicted consequences of genetic variants. Incorporating pCADD into the GWAS pipeline may help their identification. Our objective was to identify genomic regions associated with loin depth and muscle pH, and identify regions of interest for fine-mapping and further experimental work. Genotypes for ~ 40,000 single nucleotide morphisms (SNPs) were used to perform GWAS for these two traits, using de-regressed breeding values (dEBV) for 329,964 pigs from four commercial lines. Imputed sequence data was used to identify SNPs in strong ($\ge$ 0.80) linkage disequilibrium with lead GWAS SNPs with the highest pCADD scores.

**Results:**

Fifteen distinct regions were associated with loin depth and one with loin pH at genome-wide significance. Regions on chromosomes 1, 2, 5, 7, and 16, explained between 0.06 and 3.55% of the additive genetic variance and were strongly associated with loin depth. Only a small part of the additive genetic variance in muscle pH was attributed to SNPs. The results of our pCADD analysis suggests that high-scoring pCADD variants are enriched for missense mutations. Two close but distinct regions on SSC1 were associated with loin depth, and pCADD identified the previously identified missense variant within the *MC4R* gene for one of the lines. For loin pH, pCADD identified a synonymous variant in the *RNF25* gene (SSC15) as the most likely candidate for the muscle pH association. The missense mutation in the *PRKAG3* gene known to affect glycogen content was not prioritised by pCADD for loin pH.

**Conclusions:**

For loin depth, we identified several strong candidate regions for further statistical fine-mapping that are supported in the literature, and two novel regions. For loin muscle pH, we identified one previously identified associated region. We found mixed evidence for the utility of pCADD as an extension of heuristic fine-mapping. The next step is to perform more sophisticated fine-mapping and expression quantitative trait loci (eQTL) analysis, and then interrogate candidate variants in vitro by perturbation-CRISPR assays.

**Supplementary Information:**

The online version contains supplementary material available at 10.1186/s12711-023-00815-0.

## Background

Pork is one of the most consumed meats in the world, accounting for around 34% of the world’s meat consumption in 2021 [1]. Meeting consumer demand for high-quality meat in the most cost-efficient way is a major aim in pig breeding, and two traits that contribute to these goals are loin muscle depth and ultimate pH. Loin muscle depth is a measure of the amount of loin muscle in the carcass and is a key determinant of profitability. After slaughter, the pH of muscle tissue declines, as the glycogen stored in muscle is broken down into lactic acid. Ultimate pH is a measure of pH at fixed points after slaughter and a faster rate of pH decline affects the colour and water-holding capacity of the muscle fibres and is a direct cause of reduced meat quality.

Accelerating the genetic improvement of major traits by applying marker-assisted selection benefits the efficiency of pig breeding. However, dissection of the genetic architecture of these traits remains a complex and challenging task, and few definitive causal variants have been identified for any trait in pigs. A missense mutation of the *melanocortin 4 receptor* (*MC4R*) gene is involved in energy homeostasis and somatic growth [2] and also in feed efficiency in pigs [3]. Likewise, a missense mutation within the *protein kinase AMP-activated non-catalytic subunit gamma 3* (*PRKAG3*) gene located on Sus scrofa chromosome (SSC) 5 has been shown to affect the glycogen level in muscle in the Hampshire breed [4], and the effect is so large that this allele has been eliminated from many Hampshire-based commercial pig lines via marker-assisted selection. A splice mutation within the *phosphorylase b kinase gamma catalytic chain* (*PHKG1*) gene located on SSC3 has also been shown to affect glycogen content [5].

To demonstrate that variants are causative, biological assays are required. However, computational methods such as genome-wide association studies (GWAS) are an important first step in identifying candidate variants. Single marker GWAS aim at identifying the underlying causal genetic basis of a trait by independently testing each genetic variant for statistical association with a trait of interest. Whole-genome sequencing of many animals is costly and, therefore, medium-density single nucleotide polymorphism (SNP) panels are often used for GWAS in livestock. This method relies on linkage disequilibrium (LD) between the SNPs present on a panel and causative variants to identify genomic regions associated with a trait. However, high LD extends over long distances in livestock breeding populations and identifying a causative variant from the many variants that may be in high LD with a GWAS SNP remains difficult.

Pig Combined Annotation Dependent Depletion (pCADD) is an algorithm that scores genetic variants according to their predicted functional impact [6]. Derks et al. [7] incorporated pCADD scores into the GWAS pipeline and developed a simple method to identify potentially causative SNPs from sequence data. In this study, we performed a large-scale GWAS using SNP data for loin muscle depth and loin and ham pH in four intensely selected purebred pig lines. Then, we used SNPs imputed from whole-genome sequence data in a downstream pCADD analysis to identify candidate causative variants. Our main objective was to identify regions of the genome associated with loin depth and muscle pH, and to identify candidate regions of interest for fine-mapping and further experimental work.

## Methods

### Dataset

This study used breeding records on 318,964 pigs from the Pig Improvement Company (PIC; Hendersonville, TN). Pigs from two maternal lines (referred to as lines A and B) and two terminal lines (referred to as lines C and D), and from crossbred progeny sired by lines C or D were used in this analysis. The number of pigs used for each trait and line is in Table 1. Loin depth was measured in purebred and crossbred animals, while ham and loin pH were measured in crossbred animals only and attributed to the contributing breed.

**Table 1:** Number of individuals for each trait and line analysed

Trait	Line	Heritability	dEBV reliability	Number of individuals
Loin depth^a^	A	0.25	0.32	64,004
Loin depth^a^	B	0.21	0.28	55,831
Loin depth^a^	C	0.23	0.38	26,389
Loin depth^a^	D	0.36	0.45	85,833
Loin depth^b^	C	0.09	0.26	17,801
Loin depth^b^	D	0.25	0.25	63,916
Ham pH^b,c^	C	0.04	0.38	552
Ham pH^b,c^	D	0.07	0.45	1819
Loin pH^b,d^	C	0.02	0.33	817
Loin pH^b,d^	D	0.06	0.45	2002

^a^These phenotypes were measured on purebred progeny

^b^These phenotypes were measured on crossbred progeny sired by lines C and D. GWAS was performed on purebred sires

^c^pH of semimembranosus muscle measured 22 h post-slaughter

^d^pH of *longissimus* muscle measured 22 h post-slaughter

Muscle phenotypes included loin depth, measured via ultrasound at the 10th rib, and muscle pH, measured 22 h post-slaughter in the *longissimus* (loin) and *semimembranosus* (ham) muscles. Pedigree-based breeding values were estimated by genetic line with a linear mixed model that included polygenic and non-genetic effects (as relevant for each trait), as well as the dam’s breed effect in analyses of phenotypes on crossbred progeny. De-regressed breeding values (dEBV) were derived from the EBV for each trait as in [8] and used as pseudo-phenotypes in GWAS.

The breeding values, dEBV, and their reliabilities (REL) were estimated using the BLUPF90 suite programs [9]. The de-regression method is as follows:

$$dEBV=\left(\frac{EBV-PA}{R}\right)+PA,$$

where $PA$ is the parent average and $EBV$ is the individual’s EBV. $R$ is the de-regression factor obtained by:

$$R=\frac{{DE}_{individual}}{{DE}_{individual}+{DE}_{PA}+1},$$

where, following [10, 11], ${DE}_{individual}$ is the number of ‘daughter’ equivalents for the individual’s performance and its progeny and ${DE}_{PA}$ is the number of ‘daughter’ equivalents for $PA$. The ${DE}_{individual}$ and ${DE}_{PA}$ are calculated as follows:

$${DE}_{individual} =\frac{{REL}_{EBV}}{(1-{REL}_{EBV})},$$

$${DE}_{PA} = \frac{{REL}_{PA}}{(1-{REL}_{PA})},$$

where ${REL}_{EBV}$ and ${REL}_{PA}$ are the reliabilities of the individual’s EBV and of its PA, respectively.

Where relevant, a distinction is made in the text between results that were obtained from loin depth dEBV calculated based on purebred versus crossbred performance. Individuals with a dEBV reliability lower than 0.2 were excluded from further analysis.

Purebred animals were genotyped using Illumina SNP chips, with SNPs mapped to the Sscrofa11.1 genome assembly. The following SNPs were removed from further analyses: SNPs with a minor allele frequency lower than 0.01 and 0.05 for the analysis of loin depth and muscle pH, respectively, and SNPs with a call rate lower than 0.95. After quality control, an average 42,344 and 39,520 SNPs were available for each line for loin depth, and pH traits, respectively.

Variants from whole-genome sequence datasets were available from a previous study [12]. Briefly, approximately 230,000 pigs from four PIC populations (with 18,300 to 107,800 individuals per population) were genotyped using low- to medium-density SNP panels (between 15,000 and 75,000 SNPs) (GeneSeek). Approximately 2% of the animals in each population were sequenced and used to impute the remaining individuals to whole-genome sequence data with hybrid peeling using AlphaPeel [13], with an imputation accuracy of 0.97, which was evaluated by removing the sequence data of individuals with high coverage, using a leave-one-out design. The imputed variants were used in the present study to investigate causative variants post-GWAS (see ‘Identifying candidate SNPs’, below).

### Model for analysis

To account for the heterogeneous variance of the dEBV, a weight was calculated for each individual $i$ using the following formula from [14]:

$$\frac{1}{{w}_{i}}=\frac{1-{h}^{2}}{\left[c+\frac{1-{r}_{i}^{2}}{{r}_{i}^{2}}\right]{h}^{2}},$$

where ${h}^{2}$ is the line-specific heritability of the trait, ${r}^{2}$ is the reliability of the dEBV for the individual, and $c$ is the fraction of the variance not explained by markers, which was assumed to be 0.5. The reciprocal of the calculated weights was used as a residual weighting factor in the GWAS analyses, using the -widv option in the genome-wide efficient mixed-model association (GEMMA) software [15]. The GEMMA software was also used to calculate centered genomic relatedness matrices (${\mathbf{G}}_{\mathrm{c}}$) using the following formula:

$${\mathbf{G}}_{\mathrm{c}}= \frac{1}{p} \sum_{i=1}^{p}\left({\mathbf{X}}_{{\varvec{i}}}-{\bf{1}}_{n}{\overline{x} }_{i}\right){({\mathbf{X}}_{{\varvec{i}}}- {\bf{1}}_{n}{\overline{x} }_{i})}^{T},$$

where ${\mathbf{X}}_{i}$ is the *i*th column of the $n\times p$ genotype matrix $\mathbf{X}$ for $n$ individuals and $p$ SNPs, representing genotypes of the *i*th SNP, ${\overline{x} }_{i}$ is the mean genotype of the animals for SNP $i$ and ${\bf{1}}_{n}$ is an $n\times 1$ vector of 1s. The GEMMA software was also used to fit a series of univariate linear mixed models:

$$\mathbf{y}=\mathbf{X}{\varvec{\upbeta}}+\mathbf{u}+\mathbf{e},$$

where $\mathbf{y}$ is the vector of dEBV, ${\varvec{\upbeta}}$ is the vector of effects for each tested SNP, $\mathbf{X}$ is the genotype matrix, as described above, $\mathbf{u}\sim N({\bf{0}},{\mathbf{G}}_{\mathrm{c}}{\mathrm{s}}_{\mathrm{u}}^{2}$) is the vector of the polygenic additive effect with covariance matrix equal to ${\mathbf{G}}_{\mathrm{c}}$ and additive genetic variance, and $\mathbf{e}\sim N({\bf{0}},\mathbf{W}{\mathrm{s}}_{\mathrm{e}}^{2})$ is the vector of the residual errors, with diagonal matrix $\mathbf{W}$ matrix containing weights ${w}_{i}$ and residual variance ${\mathrm{s}}_{\mathrm{e}}^{2}$. To conservatively account for multiple comparisons in the GWAS, P-values were adjusted by Bonferroni correction and a SNP was declared significant at the genome or chromosome level if the − log_10_(P-value) was greater than − log_10_(0.05/*n*), where *n* represents the number of analysed SNPs across the genome, or on a given chromosome. Manhattan plots were generated using the R package CMplot.

#### Genetic variance explained by SNPs

For each trait, GWAS SNPs that showed an association at the chromosome-wide level or a higher level were combined for all genetic lines. From these combined SNPs, the significant GWAS SNPs located within 0.5 Mb of each other, plus all SNPs located within 0.5 Mb up or downstream of significant GWAS SNPs were defined as a distinct genomic region [16, 17]. To estimate the additive genetic variance for each genomic region, a ridge regression model was fitted using AlphaBayes [18]. AlphaBayes uses the same inputs as GWAS but it analyses all SNPs simultaneously to account for LD between markers within and outside the genomic regions [19]. Posterior samples of the effects for each region were obtained from 40,000 Markov-chain Monte Carlo iterations with a 5000-iteration burn-in period. For each iteration of the model, breeding values were calculated for each individual and the variance of the breeding values provided the total additive genetic variance [19]. Breeding values were also calculated for each genomic region using the SNPs located within that region. The additive genetic variance attributable to a genomic region was then calculated as the ratio of the variance of the breeding values for that region and the total additive genetic variance.

#### Identifying candidate variants and genes

To identify candidate sequence variants from the identified GWAS regions, the GWAS SNP with the lowest P-value (lead GWAS SNP) from each genomic region (as described above) was identified. Then, the PLINK v1.09 software (Boston, MA, USA) [20] was used to identify SNPs from imputed whole-genome sequence data that were in high LD (≥ 0.80) with the lead GWAS SNP. To further narrow down the list of candidate SNPs, pCADD scores were used. The pCADD tool [6] provides a score for each SNP within the pig genome based on its predicted functional impact, with a high score indicating a greater predicted functional impact. In this study, pCADD scores were obtained for all SNPs from the sequence data that were found to be in high LD with the lead GWAS SNP. The variants with the highest pCADD score associated with each lead GWAS SNP were also annotated for their variant type, using the Ensembl Variant Effect Predictor (VEP) [21], which classifies the consequences of a genomic variant on genes, transcripts, and protein sequences (e.g., synonymous, missense, etc.), and identifies genes and transcripts that are affected by the variant. To identify potential candidate genes in the identified genomic regions, variants with the top 5% of pCADD scores and their associated genes were identified using the BioMart data mining tool [22].

#### Overlap with open chromatin regions

In order to prioritize noncoding variants, we used pig muscle open chromatin sequencing data from [23-25]. We combined the peak call files of the ATAC-seq and H3K27ac ChIP-seq data for all muscle samples from these studies, i.e. 24 samples (14 ATAC-seq and 10 ChIP-seq), into one set of merged regions using the GenomicRanges package [26]. This resulted in 1.1 million open chromatin regions that covered 556 Mb of the pig genome.

## Results

**Fig. 1:**
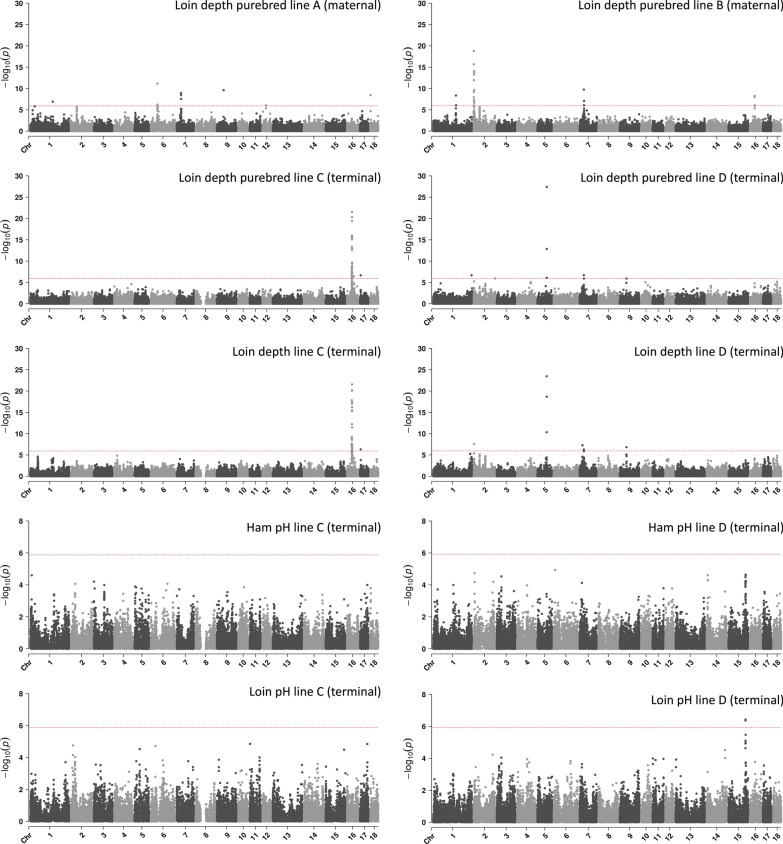
Manhattan plots showing the genome-wide associations with loin depth and with ham and loin pH in four commercial pig lines. The dotted horizonal line denotes genome-wide significance

The GWAS revealed 15 distinct genomic regions that are associated with loin muscle depth on a genome-wide level. Prioritisation of the candidate variants in these regions using pCADD identified 22 variants and 24 genes that may be responsible for these associations. Figure 1 shows the genome-wide associations by trait and line. We found one genomic region that was associated with loin muscle pH, but no associations that reached genome-wide significance for ham muscle pH. For the association with loin muscle pH, the highest-scoring pCADD variant was a synonymous SNP in the *RNF25* gene.

Table 2 summarises the main findings of the GWAS for regions that were significantly associated with traits at the genome-wide level. Regions that were significant at the chromosome-wide level are in Additional file 1: Table S1. Several regions with strong genome-wide associations with loin depth were shared by two or more genetic lines, but none of the regions were shared across all four commercial pig lines. A breakdown of the additive genetic variance explained by each genomic region is presented in Additional file 2: Tables S2–S5. Analysis of the additive genetic variance suggests that muscle traits are highly polygenic, with each identified genomic region capturing only a small amount of the total variance. For the maternal line A, 5.0% of the additive genetic variance in loin depth was captured by the identified genomic regions, whereas significant regions captured only 2.7% of the additive genetic variance for loin depth for terminal line D crossbred animals (Table 3). The amount of genetic variance captured by the identified genomic regions was much smaller for muscle pH than for loin depth, and ranged from 0.1 (ham pH, line D) to 0.7% (loin pH, line D; Table 3). The largest amount of genetic variance captured for any one genomic region was 4.0%, on SSC16 for line C and loin depth measured in purebred animals (see Additional file 2: Table S2).

**Table 2:** Summary of the genomic regions associated with each trait at a genome-wide significance threshold

Trait	SSC	Position (Mb)	Line	Number of significant SNPs	Most significant SNP		
					Position (bp)	− log_10_(P-value)	MAF
LDP	1	158.36–158.36	A	1	158,355,905	6.9	0.32
LDP	1	159.7–161.33	B	3	160,347,188	8.36	0.24
LDP	1	270.35–270.35	D	1	270,354,493	6.66	0.31
LDP	2	0.12–0.69	B	5	404,057	18.83	0.06
LDX	2	2.05–2.05	D	2	2,051,230	7.57	0.23
LDP	2	2.05–3.99	B	13	2,215,904	14.1	0.18
LDP	5	66.1–66.22	D	3	66,103,958	27.4	0.43
LDX	5	66.1–66.22	D	3	66,103,958	23.47	0.43
LDP	6	45.74–47.55	A	7	45,744,000	11.14	0.29
LDX	7	20.59–20.59	D	1	20,586,603	7.26	0.26
LDP	7	30.1–30.23	D	2	30,095,798	6.71	0.03
LDX	7	30.1–30.23	D	2	30,095,798	6.28	0.03
LDP	7	30.32–31.64	A	6	30,317,219	8.94	0.31
LDP	7	30.32–31.96	B	6	30,317,219	9.74	0.26
LDP	9	47.65–47.65	A	1	47,650,875	9.59	0.36
LDX	9	47.28–47.65	D	2	47,650,875	6.81	0.36
LDP	12	25.48–25.53	A	2	25,532,133	6.03	0.28
LDP	16	33.18–33.49	B	5	33,283,559	8.25	0.44
LDP	16	31.83–36.49	C	54	34,100,484	21.55	0.17
LDX	16	32.39–36.86	C	61	34,893,593	21.56	0.18
LDP	16	48.16–48.62	C	3	48,157,349	6.41	0.28
LDP	17	5.31–5.31	C	1	5,307,502	6.64	0.18
LDX	17	5.31–5.31	C	1	5,307,502	6.3	0.2
LDP	18	2.36–2.38	A	2	2,380,106	8.43	0.03
PHLOIN	15	119.93–121.09	D	8	120,437,865	6.42	0.23

*LDP* loin depth (purebreds), *LDX* loin depth (crossbreds), *PHHAM* pH of semimembranosus muscle measured 22 h post-slaughter, *PHLOIN* pH of longissimus muscle measured 22 h post-slaughter, *MAF* minor allele frequency

**Table 3:** Percentage of additive genetic variance explained by genomic regions with genome- and chromosome-wide significant SNP associations for loin muscle depth and muscle pH (ham and loin) in the four genetic lines evaluated

	Line A	Line B	Line C	Line D
Loin depth (purebreds)	5.0	5.6	6.1	4.3
Loin depth (crossbreds)^a^	–	–	4.2	2.7
Ham muscle pH^b^	–	–	0.1	0.1
Loin muscle pH^a,c^	–	–	0.4	0.7

^a^Although these traits were measured on crossbred individuals, GWAS was performed on purebred relatives

^b^pH of semimembranosus muscle measured 22 h post-slaughter

^c^pH of *longissimus* muscle measured 22 h post-slaughter

### Genomic regions of interest—loin depth

Fifteen distinct genomic regions were identified for loin depth, most of which have been previously reported. With the objective of identifying novel regions (i.e., regions that have not been described in the literature) and candidate causative loci, we present the two novel associations identified and the five regions that displayed the strongest GWAS associations.

*Novel regions*: A single SNP located on SSC17 (~ 5.3 Mb) was found to be associated on a genome-wide level with dEBV based on both purebred and crossbred relatives in terminal line C, while two SNPs located on SSC18 (~ 2.4 Mbp) were found for maternal line A. These associations were not detected in any of the other lines.

*Strong associations*: We identified strong associations for loin depth on SSC1, 2, 5, 7, and 16. Three SNPs on SSC1 at ~ 158.4 Mb were found for maternal line B. This region was not detected in the other lines, however a single SNP located ~ 2 Mb away from those identified in line B was identified for the maternal line A. Twenty SNPs across two regions on SSC2, at 0.4 Mb and 2.2 Mb, were identified for lines B and D. Three SNPs on SSC5 at ~ 66.1 Mb with a lead SNP − log_10_(P-value) of 27.4 were found to be associated with loin depth for line D (based on purebred and crossbred phenotypes). This region was not identified in other lines at either the genome- or chromosome-wide level. Between one and six SNPs on SSC7, spanning 1.8 Mb (30.1 to 31.96 Mb), showed associations in both maternal lines and in terminal line D based on both purebred and crossbred performance. Finally, from 5 to 61 SNPs that spanned more than 4.5 Mb (31.83 to 36.86 Mb) on SSC16 were associated with loin depth in one maternal line and in the terminal line C purebred and crossbred datasets. Another chromosome-wide level association was also found in this region in terminal line D.

### Genomic regions of interest—muscle pH

We analysed ham and loin pH in two terminal sire lines. Only one genome-wide association was found for muscle pH, on SSC15, accounting for 0.44% of the additive genetic variance. This region included eight SNPs that were significant at the genome-wide level and spanned 1.16 Mb (119.93 to 121.09 Mb). Five other regions on SSC2, 6, 11, and 17 were associated with ham or loin pH at the chromosome-wide level.

### Prioritisation of candidate variants

We used LD to define regions around each lead SNP that harboured potential candidate causative variants and genes from whole-genome sequence data. Within these regions, the variants with the highest pCADD scores were identified as potential candidate variants and the genes that contained these variants were identified as candidate genes. Table 4 shows the highest-scoring candidate gene for each region, based on pCADD scores. In many cases, pCADD identified different top candidate genes for the same region in different lines. For example, for the SSC7 region, pCADD prioritised the genes *copine 5* (*CPNE5*), *nudix hydrolase 3* (*NUDT3*), and *glutamate metabotropic receptor 4* (*GRM4*) for different lines. In total, this region includes 19 genes that contained SNPs in the top 5% of pCADD scores. Similarly, for the SSC16 region, pCADD highlighted the *ADP-ribosylation factor-like protein 15* (*ARL15*) and *chondroitin sulfate proteoglycan family member 4B* (*ENSSSCG00000016898*) genes for different lines with 18 top 5% pCADD candidate genes.

**Table 4:** Candidate variants identified for the genome-wide significant lead GWAS SNPs based on pCADD scores

Trait	SSC	Lead GWAS SNP		seqSNP							
		Position (bp)	Line	r^2^	Dist (Mb)	pCADD score	VEP	Position (bp)	Open chromatin	Gene	Supporting evidence
LDP	1	158,355,905	A	0.84	− 3.51	24.86	missense	161,864,397	No	*ENSSSCG00000004911*	-
LDP	1	160,347,188	B	0.97	− 0.43	34.19	missense	160,773,437	No	*MC4R*	Associated with growth and fatness [2]
LDP	1	270,354,493	D	0.96	− 0.05	30.69	missense	270,408,730	Yes	*HMCN2*	Differential methylation between Chinese and YP [27]
LDP	2	404,057	B	0.95	− 0.03	22.4	missense	432,824	Yes	*TMEM80*	–
LDX	2	2,051,230	D	0.81	0	20.4	missense	2,048,612	Yes	*SLC22A18*	Hypermethylated in Duroc (fat), compared to Pietrain (lean) pigs [28]
LDP	2	2,215,904	B	0.91	0	11.74	intron	2,213,982	No	*OSBPL5*	Candidate gene for ADG [29]
LDP	5	66,103,958	D	0.98	− 0.01	13.49	intron	66,109,890	Yes	*CCND2*	Overexpressed in SM muscle [30]
LDX	5	66,103,958	D	0.98	− 0.01	13.49	intron	66,109,890	Yes	*CCND2*	Overexpressed in SM muscle [30]
LDP	6	45,744,000	A	0.86	− 6.29	34.95	missense	52,032,079	Yes	*GIPR*	–
LDX	7	20,586,603	D	0.91	0.28	9.91	intron	20,305,193	No	*CARMIL1*	Suggested gene for backfat GWAS [31]
LDP	7	30,095,798	D	0.94	− 0.08	14.82	missense	30,179,826	Yes	*GRM4*	eQTL for fatty acid composition [32]
LDX	7	30,095,798	D	0.94	− 0.08	14.82	missense	30,179,826	Yes	*GRM4*	eQTL for fatty acid composition [32]
LDP	7	30,317,219	A	0.88	− 2.11	28.78	synonymous	32,427,677	No	*CPNE5*	Candidate gene for muscle lightness [33]
LDP	7	30,317,219	B	0.87	− 0.03	17.58	3 prime UTR	30,350,337	Yes	*NUDT3*	Associated with loin muscle area [34, 35]
LDP	9	47,650,875	A	0.96	0.18	11.79	intron	47,475,749	Yes	*ARHGEF12*	
LDX	9	47,650,875	D	0.92	0.24	7.22	intron	47,406,822	No	*POU2F3*	–
LDP	12	25,532,133	A	0.93	0.23	34.62	missense	25,299,992	No	*ENSSSCG00000030269*	–
LDP	16	33,283,559	B	0.92	0.09	17.4	intron	33,194,036	No	*ARL15*	Candidate gene for growth [36]
LDP	16	34,100,484	C	0.96	0.05	43.19	missense	34,048,944	No	*ENSSSCG00000016898*	–
LDX	16	34,893,593	C	0.9	0.84	43.19	missense	34,048,944	No	*ENSSSCG00000016898*	–
LDP	16	48,157,349	C	0.84	− 3.7	21.66	synonymous	51,852,935	Yes	*SH3PXD2B*	Implicated in growth rate of pigs [37]
LDP	17	5,307,502	C	0.91	0.06	34.21	synonymous	5,246,513	Yes	*SLC7A2*	–
LDX	17	5,307,502	C	0.91	0.06	34.21	synonymous	5,246,513	Yes	*SLC7A2*	–
LDP	18	2,380,106	A	0.91	− 0.04	23.47	intergenic	2,422,486	No	*ENSSSCG00000042976*	–
PHLOIN	15	120,437,865	D	0.97	− 0.28	41.82	synonymous	120,713,562	Yes	*RNF25*	Suggested role in muscle development in cattle [38]

*seqSNP* the SNP with a top ranking pCADD score from sequence data, *LDP* loin depth (purebreds), *LDX* loin depth (crossbreds), *PHHAM* pH of semimembranosus muscle measured 22 h post-slaughter, *PHLOIN* pH of *longissimus* muscle measured 22 h post-slaughter, *seqSNP* SNP with the highest pCADD score in high LD (r^2^ > 0.07) with the lead GWAS SNP. r^2^: linkage disequilibrium between lead GWAS SNP and seqSNP; Distance: distance in Mbp between lead GWAS SNP and seqSNP; VEP = Ensembl variant effect prediction of the seqSNP; Position of the seqSNP; Gene refers to the gene each seqSNP was located within, or the closest gene if the seqSNP was intergenic

Table 4 shows the SNPs with a top ranking pCADD score (seqSNP) for each genome-wide significant lead GWAS SNP, along with the predicted effect for each SNP, and its distance from, and LD with the lead GWAS SNP (for results on regions that were significant at the chromosomal level, see Additional file 3: Table S6). Candidate variant prioritisation by pCADD identified primarily protein coding variants for the significant associations, which are often located far away (on average 566 kb, calculated from the data in Table 4) from the most significant SNP. Among the variants that were identified in exons, 11 were missense variants, five were synonymous, and one was a UTR. For loin depth, pCADD identified a missense mutation in the *MC4R* gene as a candidate variant in maternal line B. For the region on SSC7 that was shared between the terminal line D (purebred and crossbred phenotypes) and both maternal lines, the top pCADD scores were found for variants located in different genes, i.e. *CPNE5*, *NUDT3*, and *GRM4.*

For 10 of the 29 line-specific regions that were found to be associated with loin depth or pH at the genome-wide level, none of the SNPs in high LD with the lead SNP contained missense mutations. In the other 19 regions, only 0.72% of all SNPs that were in high LD with the lead GWAS SNP were missense variants, while approximately 9.6% of these SNPs were in the top 5% of pCADD scores. Sixty eight percent of all missense variants that were in high LD with lead GWAS SNPs appeared in the top 5% of variants identified by pCADD, while 38% of the top scoring variants identified by pCADD were missense mutations.

**Fig. 2:**
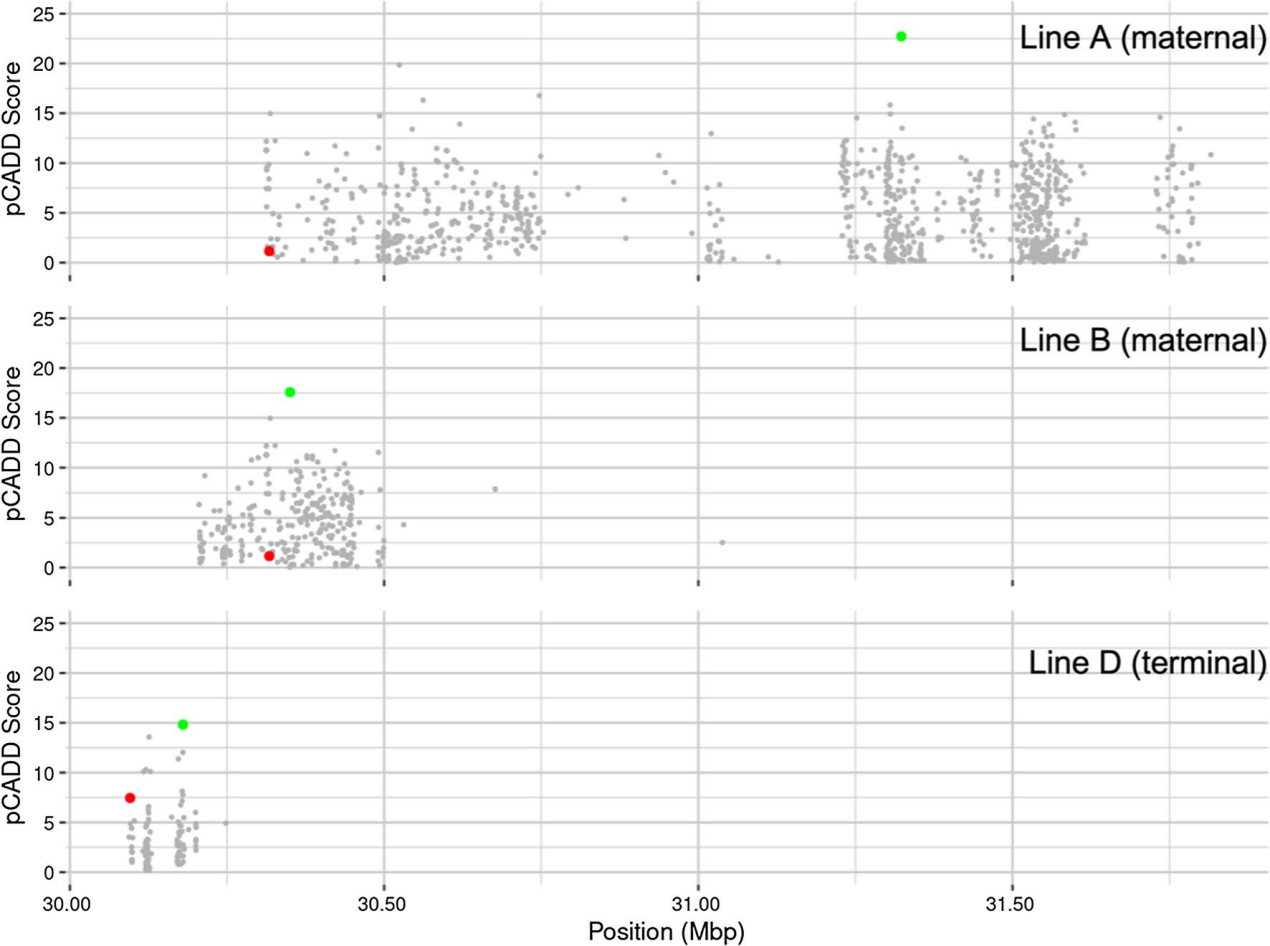
Plot showing the sequence SNPs (grey) that are in high linkage disequilibrium (r^2^ > 0.80) with the lead GWAS SNP (red) from the region on SSC7 that was found to be associated with loin depth in three commercial pig lines. The candidate causal variant identified based on pCADD scores is highlighted in green

To explore whether the combination of LD and pCADD analyses are sufficiently accurate to prioritise variants for further functional studies, we looked more closely at the main regions that were found to be associated with loin depth in the current study. The region on SSC7 that was identified in three of the genetic lines was also highlighted by Derks et al. [7], who used a similar methodology and this, therefore, provides a useful comparison. Figure 2 shows the position and pCADD score of the seqSNP, and all the SNPs that are in high LD with the GWAS SNP for each of these three genetic lines.

## Discussion

### Identification of novel associations

#### Loin muscle depth

We found two previously unreported regions on SSC17 and 18 to be associated with loin muscle depth. The variants identified by pCADD in these regions were a synonymous variant located in the *SLC7A2* gene (SSC17) and an intergenic variant (SSC18). The *SLC7A2* gene encodes an amino acid transporter that has been shown to be downregulated in pigs on severely protein-restricted diets [39] and is therefore a highly plausible candidate gene for loin muscle traits.

### Loin and ham muscle pH

We did not identify novel regions that were significantly associated with loin or ham pH at the genome-wide level. Although similar population sizes were used as in e.g. [39], we failed to replicate many GWAS findings reported in the literature, which may be explained by the relatively small population size, combined with the low average reliability of dEBV in our study. Raw phenotypes were not available for this analysis, but they could be considered as an alternative to dEBV in other studies.

### Replication and refinement of previously known genomic regions for muscle and growth traits

A region spanning 1.16 Mb on SSC15 was found to be associated with ham pH at the genome-wide level and this region includes the *protein kinase AMP-activated non-catalytic subunit gamma 3* (*PRKAG3*) gene. While relatively few mutations have definitively been associated with pork meat quality traits [40], two mutations in the *PRKAG3* gene have been shown to increase glycogen level in the muscle of pigs. Pigs carrying these mutations produce inferior meat with a lower pH (so-called “acid meat”). The previously identified missense mutation in the *PRKAG3* gene was the 7th highest-scoring pCADD SNP, while the highest scoring variant was a synonymous variant located in the *ring finger protein 25* (*RNF25)* gene. This gene encodes a ligase that partly controls *naked cuticle 2 (NKD2) *degradation in humans [38], which has been suggested to have a role in muscle development [41]*.* Although *RNF25* may have a role in muscle pH, the more likely candidate causative gene for the association with muscle pH identified in this region is *PRKAG3*.

Our study replicated five regions on SCC1, 2, 5, and 16 that were strongly associated with loin muscle depth and that have previously been shown to be associated with muscle and growth traits and prioritised potential candidate causative variants for these regions. In the following, we briefly discuss the overlaps between the genes reported in the literature and the genes that we identified here using pCADD prioritisation.

#### SSC1

The region identified on SSC1 has been detected in several GWAS for loin depth and backfat [7, 17, 42, 43]. In our study, the lead GWAS SNP was located 0.4 Mb downstream from the *MC4R* gene and the highest-scoring pCADD variant was a missense variant located within this gene. Several mutations in the pig *MC4R* gene are well known and are involved in regulating appetite [44, 45]; the variant that we identified has also been described to affect fatness, growth, and feed intake in different pig breeds [2, 46, 47]. Given the supportive evidence from multiple previous studies and the plausible biological pathways in which *MC4R* is involved, the pCADD-identified missense mutation in *MC4R* and in the regions around the *MC4R* gene represent strong candidates for effects on muscle loin depth in pigs and warrant further fine-mapping to identify other causative variants.

#### SSC2

Two regions on SSC2 were strongly associated with loin depth, one from 0.12 to 0.69 Mb and the other from 2.05 to 3.99 Mb. These were treated as separate regions in our analysis since there was an interval of more than 0.5 Mb between them, however Gozalo-Marcilla et al. [17] found one large association with backfat that spanned this entire region, accounting for 6.7% of the additive genetic variance. In our study, these regions jointly accounted for 2.8% of the additive genetic variance in line B. This is one of several regions identified in our study that overlaps with regions that were previously shown to be associated with backfat, which may be due to the negative genetic correlations between loin depth and backfat [48]. An intronic variant in the *oxysterol binding protein like 5* (*OSBPL5*) gene was identified as a candidate gene for this region. In humans, *OSBPL5* plays a key role in the maintenance of cholesterol balance in the body [49], and thus represents a plausible candidate for body composition traits in pigs.

#### SSC5

The region identified on SSC5 was previously reported to be associated with backfat [7, 17], average daily gain [50], and meat tenderness [51]. In our study, pCADD prioritisation identified *cyclin D2* (*CCND2*) as a candidate gene. The *CCND2* gene is important for the growth of pancreatic islets and is involved in hormonal regulation of growth [52], has recently been fine-mapped as a candidate gene for backfat [53] and for conformation traits [52] in pigs, and has been found to be overexpressed in the *semimembranosus* muscle of pigs [30]. Thus, *CCND2* is a plausible candidate gene for the regulation of loin muscle traits in pigs.

#### SSC16

The region that explained the largest proportion of genetic variance in loin depth was located on SSC16. Several studies have detected markers within this region that are associated with loin muscle [35, 54, 55], feed conversion rate [56], backfat [17, 57], and growth [36, 58], but no causative SNPs have been confidently proposed. Bergamaschi et al. [55] found that this region explained more than 4% of the variance in loin muscle depth and the gene network analysis performed in that study suggested *GPX8* as a candidate gene, but this gene was not identified in the top 5% of pCADD scores in our study. Instead, the variants identified by pCADD in this region were a synonymous mutation in the *SH3 and PX domains 2B* (*SH3PXD2B*) gene, an intronic variant in the *ADP ribosylation factor like GTPase 15* (*ARL15*) gene, and a missense variant in an uncharacterised protein coding gene. Since this genomic region contains 70 genes (results not presented), it is difficult to pinpoint any one of the genes as causative using the pCADD methodology. However, the strength of this association and its replicability make this a strong candidate region for detecting causative variants via fine-mapping and biological assays.

### The utility of linkage disequilibrium and pCADD scores for fine-mapping complex traits

The prioritisation scheme of LD in combination with pCADD scores identified several candidate causative variants in genes that have plausible biological links to muscle. The question is whether combining LD and pCADD is sufficiently accurate to prioritise variants for further functional studies. To explore this, we looked more closely at the main regions that were identified to be associated with loin depth in our study. One region on SSC7 that was detected in three of the genetic lines was also highlighted by Derks et al. [7], who used a similar methodology, which allows a relevant comparison between the results of these two studies.

Derks et al. [7] reported that this region is associated with backfat, intramuscular fat, growth rate, and drip loss in Duroc pigs, and the candidate variant that was identified by pCADD was located in the *HMGA1* gene, which has been implicated in growth and carcass traits in pigs [59]. The candidate mutation that was reported by Derks et al. [7] was in high LD with the lead SNP in both of our maternal populations but ranked 3rd and 33rd based on our pCADD scores. In our study, the pCADD scores highlighted variants in the *GRM4*, *CPNE5*, and *NUDT3* genes as the most likely candidates in the SSC7 region.

The two maternal lines (top two panels in Fig. 2) shared a lead GWAS SNP but the seqSNP was different for each line due to different LD patterns in the two populations. The genes in which the SNPs for these lines are positioned, *NUDT3* and *CPNE5*, respectively, are located more than 2 Mb from each other. Among the three lines presented in Fig. 2, maternal line A had the slowest rate of LD decay with distance (results not presented) and, therefore, the pool of candidate SNPs for pCADD was much larger for this line and covered a larger region. This explains why the seqSNP for this line was located more than 2 Mb away from the lead GWAS SNP, in contrast to the seqSNPs identified in lines B and D, and demonstrates how population structure can influence the candidate SNP identified using this methodology. Thus, the ability of pCADD to identify causative variants could be improved by adding a physical distance constraint, to mitigate the effect of large LD blocks. Bayesian fine-mapping of these regions may help further refine the parameters used to identify causative variants using pCADD scores.

### Noncoding genetic variants may be important contributors to complex traits

In the current analysis, 38% of the top variants identified by pCADD were missense mutations. The CADD methodology was developed based on simulated mutations to identify variants that do not segregate at high frequency, [6, 60] and, thus, tends to prioritise potentially deleterious variants with protein-coding consequences and to score noncoding variants with a lower value. Thus, we hypothesise that this 38% value likely overestimates the fraction of causative variants that are protein-coding.

Among the highest scoring SNPs identified by pCADD, one was in an intergenic region, seven were intronic, and one was located within a UTR, which suggests that the causative variants detected may be noncoding. For such regions, prioritisation needs to consider open chromatin data from muscle and, in our study, we combined such data from several studies [23-25]. We found that 14 of the variants were in regions of open chromatin obtained from muscle tissue, while 11 were not. For noncoding variants, bioinformatic prediction alone is likely not sufficient to prioritise causative variants. Candidate variants prioritised by pCADD and overlapping active chromatin in muscle could be tested for gene regulation functions in cell-based assays such as CRISPR interference or massively parallel reporter assays e.g. [61].

### Future work

Genome editing via CRISPR/Cas9 technologies has the potential to quickly and cost-effectively improve traits that are difficult to target via traditional selection methods. However, the polygenic architecture of the traits studied here, with the strongest associations explaining at most 3.6% of the additive genetic variance, suggests that the identification of candidate variants with large effects for gene editing, which needs significant investment, is unlikely.

Alternatively, variants identified from GWAS and whole-genome sequencing could be incorporated into genomic prediction models to improve their accuracy, or their ability to predict breeding values across populations, making the accuracy of genomic predictions less sensitive to differences in LD within and between populations. The most promising strategy for genomic prediction with sequence variants seems to be to add preselected variants from some combination of GWAS and functional annotation to SNP chips, rather than use millions of sequence variants directly for prediction. For example, Xiang et al. [62, 63] used GWAS and functional genomics data to identify 80k potentially causative SNPs and to develop a medium-density array for use in genomic selection in dairy cattle. However, in pigs, the strategy of pre-selecting variants from GWAS to improve genomic prediction has shown inconsistent results across traits and populations [64]. Perhaps these strategies can be improved by statistical fine-mapping and functional genomic prioritization of variants [65], if such approaches could improve the enrichment of genuine causative variants among the pre-selected variants.

## Conclusions

In this paper, we detected novel associations for loin depth and muscle pH in the pig and confirmed several previously known associations for loin depth. We identified plausible candidate genes based on whole-genome sequence data and bioinformatic variant effect prediction with pCADD, including genes involved in adipogenesis, fatty acid metabolism, and insulin signalling. Taken together with the overlap of the associated regions with backfat, this is consistent with the shared genetic basis of loin depth and backfat. However, the identified regions contain many genes and putative functional variants in high LD with each other, and the prioritised genes differed between lines due to variable LD patterns. Thus, there is considerable uncertainty in the current prioritisation of genes and variants and statistical fine-mapping in combination with empirical assays of variant function may be necessary to get closer to the causative variants.

## Supplementary Information

**Additional file 1: Table S1.** Summary of genomic regions associated with each trait at the chromosome-wide significance threshold. GWAS results significant at the chromosome-wide level

**Additional file 2: Table S2.** Percentage of additive genetic variance explained by each genomic region in four purebred pig lines. Percentage of additive genetic variance attributable to SNPs for loin depth. **Table S3.** Percentage of additive genetic variance explained by each genomic region for loin depth phenotypes calculated based on crossbred performance, in two purebred pig lines. **Table S4.** Percentage of additive genetic variance explained by each genomic region for ham pH in two purebred pig lines. **Table S5.** Percentage of additive genetic variance explained by each genomic region for loin pH in two purebred pig lines

**Additional file 3: Table S6.** List of candidate variants for chromosome-wide significant lead GWAS SNP identified using pCADD scores. Top pCADD SNP for each lead GWAS SNP found to be significant at the chromosome wide level [66-72].

## Data Availability

AlphaBayes and AlphaPeel are available from Github (https://github.com/AlphaGenes). pCADD scores are available via the Wageningen University Bioinformatics group: (https://www.bioinformatics.nl/pCADD/indexed_pPHRED-scores/). The datasets generated and analysed in this study are derived from the PIC breeding programme and not publicly available.
