# Supplementary material for: A genome-wide association study for loin depth and muscle pH in pigs from intensely selected purebred lines
Source: Genet Sel Evol. 2023 Jun 15;55:42. doi: 10.1186/s12711-023-00815-0 (PMC10268370; doi:10.1186/s12711-023-00815-0)
Supplement: Supplementary file 1 — Additional file 1: Table S1. Summary of genomic regions associated with each trait at the chromosome-wide significance threshold. GWAS results significant at the chromosome-wide level [file 12711_2023_815_MOESM1_ESM.docx]

**Table S1. Summary of genomic regions associated with each trait at a chromosome-wide significance threshold**

|  |  |  |  |  | **Most significant SNP** | | |
| --- | --- | --- | --- | --- | --- | --- | --- |
| **Trait** | **SSC** | **Position (Mb)** | **Line** | **No. significant SNPs** | **Position (bp)** | **-log10 P-value** | **MAF^1^** |
| LDP | 1 | 30.82-30.82 | A | 1 | 30816637 | 5.81 | 0.09 |
| LDP | 2 | 148.81-148.81 | D | 1 | 148814990 | 5.91 | 0.09 |
| LDP | 2 | 2.05-2.05 | D | 1 | 2051230 | 5.23 | 0.24 |
| LDP | 2 | 41.4-41.67 | B | 6 | 41570652 | 5.64 | 0.32 |
| LDP | 2 | 41.79-41.99 | A | 3 | 41860911 | 5.64 | 0.44 |
| LDP | 4 | 95.08-95.1 | D | 2 | 95095905 | 5.07 | 0.42 |
| LDP | 7 | 50.68-50.68 | B | 1 | 50682389 | 4.85 | 0.08 |
| LDP | 9 | 47.28-47.65 | D | 2 | 47650875 | 5.91 | 0.37 |
| LDP | 10 | 30.25-30.25 | D | 1 | 30250539 | 5.02 | 0.04 |
| LDP | 12 | 3.92-3.92 | A | 1 | 3917034 | 4.57 | 0.16 |
| LDP | 16 | 32.56-32.56 | D | 1 | 32564089 | 4.77 | 0.45 |
| LDP | 17 | 15.83-15.83 | A | 1 | 15827454 | 4.66 | 0.48 |
| LDP | 18 | 14.43-14.43 | D | 1 | 14429619 | 4.48 | 0.5 |
| LDP | 18 | 27.04-27.46 | D | 2 | 27039044 | 5.14 | 0.31 |
| LDX | 1 | 260.38-260.9 | D | 4 | 260869133 | 5.29 | 0.01 |
| LDX | 2 | 41.79-41.86 | D | 2 | 41860911 | 5.08 | 0.19 |
| LDX | 4 | 19.39-19.39 | C | 1 | 19389682 | 4.82 | 0.45 |
| LDX | 8 | 67.7-67.7 | D | 1 | 67695280 | 4.77 | 0.28 |
| LDX | 16 | 37.99-37.99 | C | 1 | 37994317 | 4.93 | 0.05 |
| LDX | 17 | 37.19-37.19 | D | 1 | 37194701 | 4.49 | 0.32 |
| LDX | 18 | 27.46-27.46 | D | 1 | 27462020 | 4.82 | 0.19 |
| PHHAM | 6 | 10.24-10.24 | D | 1 | 10235192 | 4.93 | 0.34 |
| PHLOIN | 2 | 16.29-16.29 | C | 1 | 16285939 | 4.75 | 0.48 |
| PHLOIN | 6 | 32.18-32.18 | C | 1 | 32177925 | 4.72 | 0.13 |
| PHLOIN | 11 | 3.11-3.11 | C | 1 | 3105902 | 4.85 | 0.06 |
| PHLOIN | 17 | 51.96-51.96 | C | 1 | 51964294 | 4.84 | 0.19 |

*LDP: loin depth (purebreds); LDX: loin depth (crossbreeds); PHHAM: pH of semimembranosus muscle measured 22 h post-slaughter; PHLOIN: pH of longissimus muscle measured 22 h post-slaughter; LACTATE: muscle lactate; IMF: intramuscular fat. MAF = Minor allele frequency*
