# Supplementary material for: A genome-wide association study for loin depth and muscle pH in pigs from intensely selected purebred lines
Source: Genet Sel Evol. 2023 Jun 15;55:42. doi: 10.1186/s12711-023-00815-0 (PMC10268370; doi:10.1186/s12711-023-00815-0)
Supplement: Supplementary file 2 — Additional file 2: Table S2. Percentage of additive genetic variance explained by each genomic region in four purebred pig lines. Percentage of additive genetic variance attributable to SNPs for loin depth. Table S3. Percentage of additive genetic variance explained by each genomic region for loin depth phenotypes calculated based on crossbred performance, in two purebred pig lines. Table S4. Percentage of additive genetic variance explained by each genomic region for ham pH in two purebred pig lines. Table S5. Percentage of additive genetic variance explained by each genomic region for loin pH in two purebred pig lines [file 12711_2023_815_MOESM2_ESM.docx]

Table S2. Percentage of additive genetic variance explained by each genomic region for loin depth in four purebred pig lines

|  |  | Maternal | | Terminal | |
| --- | --- | --- | --- | --- | --- |
| SSC | Genomic region | Line A - | Line B - | Line C- | Line D - |
| 1 | 30.32-31.32 | ***0.06*** | 0.04 | 0.06 | 0.14 |
| 1 | 157.86-158.86 | ***0.06*** | 0.03 | 0.03 | 0.02 |
| 1 | 159.2-161.83 | ***0.29*** | ***0.52*** | 0.12 | 0.20 |
| 1 | 269.85-270.85 | 0.07 | 0.04 | 0.04 | ***0.19*** |
| 2 | 0.00-1.19 | 0.02 | ***0.68*** | 0.00 | 0.02 |
| 2 | 1.55-4.49 | 0.23 | ***1.66*** | 0.16 | ***0.40*** |
| 2 | 40.9-42.49 | ***0.54*** | ***0.38*** | 0.12 | 0.24 |
| 2 | 148.31-149.31 | 0.10 | 0.07 | 0.10 | ***0.08*** |
| 4 | 94.58-95.6 | 0.07 | 0.04 | 0.09 | ***0.27*** |
| 5 | 65.6-66.72 | 0.08 | 0.03 | 0.08 | ***0.52*** |
| 6 | 45.24-48.05 | ***0.75*** | 0.07 | 0.15 | 0.24 |
| 7 | 29.6-32.46 | ***0.81*** | ***0.47*** | 0.21 | ***0.20*** |
| 7 | 50.18-51.18 | 0.08 | ***0.05*** | 0.08 | 0.02 |
| 9 | 46.78-48.15 | ***0.27*** | 0.06 | 0.06 | ***0.31*** |
| 10 | 29.75-30.75 | 0.04 | 0.10 | 0.05 | ***0.04*** |
| 12 | 3.42-4.42 | ***0.05*** | 0.03 | 0.05 | 0.04 |
| 12 | 24.98-26.03 | ***0.11*** | 0.14 | 0.05 | 0.05 |
| 16 | 31.33-36.99 | 0.63 | ***0.94*** | ***4.04*** | ***0.65*** |
| 16 | 47.66-49.12 | 0.15 | 0.04 | ***0.16*** | 0.04 |
| 17 | 4.81-5.81 | ***0.06*** | 0.04 | ***0.14*** | 0.14 |
| 17 | 15.33-16.33 | ***0.37*** | 0.03 | 0.11 | 0.14 |
| 18 | 1.86-2.88 | ***0.09*** | 0.07 | 0.08 | 0.07 |
| 18 | 13.93-14.93 | 0.05 | 0.03 | 0.02 | ***0.12*** |
| 18 | 26.54-27.96 | 0.04 | 0.03 | 0.05 | ***0.16*** |
| Remainder |  | 93.66 | 95.07 | 93.95 | 94.92 |

*Percentages in bold italics indicate lines in which each genomic region was significantly associated with loin depth*

Table S3. Percentage of additive genetic variance explained by each genomic region for loin depth phenotypes calculated based on crossbred performance, in two purebred pig lines

| **SSC** | **Genomic region (Mb)** | **Line C -** | **Line D -** |
| --- | --- | --- | --- |
| 1 | 260.38-260.9 | 0.07 | ***0.08*** |
| 2 | 2.05-2.05 | 0.02 | ***0.20*** |
| 2 | 41.79-41.86 | 0.07 | ***0.18*** |
| 4 | 19.39-19.39 | ***0.14*** | 0.07 |
| 5 | 66.1-66.22 | 0.07 | ***0.43*** |
| 7 | 20.59-20.59 | 0.06 | ***0.13*** |
| 7 | 30.1-30.23 | 0.11 | ***0.10*** |
| 8 | 67.7-67.7 | 0.00 | ***0.05*** |
| 9 | 47.28-47.65 | 0.07 | ***0.24*** |
| 16 | 32.39-36.86 | ***3.31*** | 0.91 |
| 16 | 37.99-37.99 | ***0.02*** | 0.02 |
| 17 | 37.19-37.19 | 0.11 | ***0.14*** |
| 17 | 5.31-5.31 | ***0.09*** | 0.06 |
| 18 | 27.46-27.46 | 0.03 | ***0.06*** |
| Remainder |  | ***95.13*** | 96.09 |

*Percentages in bold italics indicate lines in which each genomic region was significantly associated with loin depth*

Table S4. Percentage of additive genetic variance explained by each genomic region for ham pH in two purebred pig lines

| **SSC** | **Genomic region (Mb)** | **Line C -** | **Line D -** |
| --- | --- | --- | --- |
| 6 | 10.24-10.24 | 0.08 | ***0.08*** |
| Remainder |  | 99.94 | 9.72 |

*Percentages in bold italics indicate lines in which each genomic region was significantly associated with ham pH*

Table S5. Percentage of additive genetic variance explained by each genomic region for loin pH in two purebred pig lines

| **SSC** | **Genomic region (Mb)** | **Line C -** | **Line D -** |
| --- | --- | --- | --- |
| 2 | 16.29-16.29 | ***0.08*** | 0.07 |
| 6 | 32.18-32.18 | ***0.07*** | 0.07 |
| 11 | 3.11-3.11 | ***0.08*** | 0.05 |
| 15 | 119.93-121.09 | 0.12 | ***0.44*** |
| 17 | 51.96-51.96 | ***0.09*** | 0.04 |
| Remainder |  | 98.53 | 98.34 |

*Percentages in bold italics indicate lines in which each genomic region was significantly associated with loin pH*
