# Supplementary material for: A genome-wide association study for loin depth and muscle pH in pigs from intensely selected purebred lines
Source: Genet Sel Evol. 2023 Jun 15;55:42. doi: 10.1186/s12711-023-00815-0 (PMC10268370; doi:10.1186/s12711-023-00815-0)
Supplement: Supplementary file 3 — Additional file 3: Table S6. List of candidate variants for chromosome-wide significant lead GWAS SNP identified using pCADD scores. Top pCADD SNP for each lead GWAS SNP found to be significant at the chromosome wide level [66-72]. [file 12711_2023_815_MOESM3_ESM.docx]

**Table S6 List of candidate variants for chromosome-wide significant lead GWAS SNP identified using pCADD scores**

|  |  | **Lead GWAS SNP** | | **seqSNP** | | | | | | | |
| --- | --- | --- | --- | --- | --- | --- | --- | --- | --- | --- | --- |
| **Trait** | **SSC** | **Position (Bp)** | **Line** | **R^2^** | **Distance (Mb)** | **pCADD score** | **VEP** | **Position (bp)** | **Open chromatin** | **Gene** | **Supporting evidence** |
| LDP | 1 | 30816637 | A | 0.98 | 0.00 | 18.44 | intergenic | 30820105 | Yes | ENSSSCG00000045624 | - |
| LDX | 1 | 260869133 | D | 0.88 | -0.6 | 40.68 | Stop retained | 261469070 | No | ENSSSCG00000038200 | - |
| LDP | 2 | 2051230 | D | 0.81 | 0.00 | 20.4 | missense | 2048612 | Yes | SLC22A18 | Hypermethylated in Duroc (fat), compared to Pietrain (lean) pigs [28] |
| LDP | 2 | 41570652 | B | 0.86 | -0.42 | 38.33 | synonymous | 41994631 | No | PIK3C2A | Upregulated in high fat pigs [67]; downregulated in highly feed efficient pigs [68] |
| LDP | 2 | 41860911 | A | 0.99 | -0.13 | 38.33 | synonymous | 41994631 | No | PIK3C2A | Upregulated in high fat pigs [67]; downregulated in highly feed efficient pigs [68] |
| LDX | 2 | 41860911 | D | 0.98 | -0.13 | 38.33 | synonymous | 41994631 | No | PIK3C2A | Upregulated in high fat pigs [67]; downregulated in highly feed efficient pigs [68] |
| LDP | 2 | 148814990 | D | 0.91 | 0.04 | 12.87 | intron | 148775988 | Yes | DPYSL3 | - |
| LDX | 4 | 19389682 | C | 0.87 | -0.03 | 10.36 | intron | 19424506 | No | ENPP2 | Found to affect the differentiation of subcutaneous adipocytes in Yorkshire pigs [69]; Associated with ﻿fatty acid composition in intramuscular fat in pigs [58] |
| LDP | 4 | 95095905 | D | 0.82 | -0.82 | 38.44 | synonymous | 95913006 | Yes | INTS3 | - |
| LDP | 7 | 50682389 | B | 0.91 | 0.05 | 11.51 | intergenic | 50633098 | No | ENSSSCG00000044046 | - |
| LDX | 8 | 67695280 | D | 0.83 | -3.55 | 23.68 | missense | 71246853 | No | CDKL2 | Associated with feed efficiency traits in pigs [70] |
| LDP | 9 | 47650875 | D | 0.92 | 0.24 | 7.22 | intron | 47406822 | No | POU2F3 | - |
| LDP | 10 | 30250539 | D | 0.83 | -0.75 | 25.76 | missense | 31002152 | Yes | C9orf64 | - |
| LDP | 12 | 3917034 | A | 0.81 | 0.01 | 16.55 | missense | 3910402 | No | TNRC6C | DDX5 implicated in muscle cell differentiation [71] |
| LDP | 16 | 32564089 | D | 0.84 | 0.32 | 25.18 | missense | 32243273 | No | ITGA1 | Downregulated in highly feed efficient pigs [68]; |
| LDX | 16 | 37994317 | C | 0.83 | 3.82 | 24.91 | missense | 34175603 | No | ESM1 | Involved in cell proliferation, differentially expressed in landrace piglet adipose tissue during preadipocyte differentiation [72] |
| LDP | 17 | 15827454 | A | 0.88 | 0.29 | 23.7 | intergenic | 15542095 | No | ENSSSCG00000047884 | - |
| LDX | 17 | 37194701 | D | 0.93 | -0.01 | 23.68 | missense | 37205667 | No | ENSSSCG00000034625 | - |
| LDP | 18 | 14429619 | D | 0.9 | -0.02 | 16.97 | 5` UTR | 14451963 | Yes | CALD1 | Involved in smooth muscle contraction but CALD1 knockout pigs developed normally [73] |
| LDP | 18 | 27039044 | D | 0.83 | 0.00 | 20.32 | intergenic | 27040051 | No | ENSSSCG00000048136 | - |
| LDX | 18 | 27462020 | D | 0.88 | -0.18 | 14.14 | intergenic | 27643978 | No | ENSSSCG00000048536 | - |
| PHLOIN | 2 | 16285939 | C | 0.8 | -0.46 | 14.62 | intergenic | 16744446 | No | ENSSSCG00000045375 | - |
| PHHAM | 6 | 10235192 | D | 0.95 | -0.02 | 15.4 | intergenic | 10251888 | No | ADAMTS18 | - |
| PHLOIN | 6 | 32177925 | C | 0.8 | -0.02 | 20.34 | intergenic | 32195230 | No | ENSSSCG00000043977 | - |
| PHLOIN | 11 | 3105902 | C | 0.84 | 0.26 | 24.27 | missense | 2850902 | Yes | ENSSSCG00000009293 | - |
| PHLOIN | 17 | 51964294 | C | 0.92 | 0.01 | 13.2 | intergenic | 51957214 | No | ENSSSCG00000045060 | - |

seqSNP: the SNP with a top ranking pCADD score from sequence data; *LDP: loin depth (purebreds); LDX: loin depth (crossbreed performance); PHHAM: pH of semimembranosus muscle measured 22 h post-slaughter; PHLOIN: pH of longissimus muscle measured 22 h post-slaughter; seqSNP: SNP with the highest pCADD score in high linkage-disequilibrium > 0.07) with the lead GWAS SNP. R^2^ = linkage-disequilibrium between lead GWAS SNP and seqSNP; Distance: Distance in MB between lead GWAS SNP and seqSNP; VEP = Ensembl variant effect predictor of the seqSNP; Position of the seqSNP; Gene refers to the gene each seqSNP was located within (if any)*
